# Is the Blood Oxygenation Level-Dependent fMRI Response to Motor Tasks Altered in Children After Neonatal Stroke?

**DOI:** 10.3389/fnhum.2020.00154

**Published:** 2020-04-29

**Authors:** Mariam Al Harrach, François Rousseau, Samuel Groeschel, Stéphane Chabrier, Lucie Hertz-Pannier, Julien Lefevre, Mickael Dinomais

**Affiliations:** ^1^Laboratoire Angevin de Recherche en Ingénierie des Systèmes (LARIS) EA7315, Université d’Angers, Polytech Angers, Angers, France; ^2^INSERM U1101 LaTIM, UBL, IMT Atlantique, Brest, France; ^3^Department of Child Neurology, Paediatric Neuroimaging, University Hospital, Tübingen, Germany; ^4^INSERM UMR1059 Sainbiose, Univ Saint-Étienne, Univ Lyon, Saint-Étienne, France; ^5^INSERM, CIC 1408, CHU Saint-Étienne, French Centre for Paediatric Stroke, Paediatric Physical and Rehabilitation Medicine Department, Saint-Étienne, France; ^6^INSERM U114 Neurospin, UNIACT, Institut Joliot, Université de Paris, CEA-Paris Saclay, Gif sur Yvette, France; ^7^UMR CNRS 7289, Aix Marseille Université, Institut de Neurosciences de la Timone, Marseille, France; ^8^CHU Angers, Département de Médecine Physique et de Réadaptions and LUNAM, Angers, France

**Keywords:** neonatal arterial ischemic stroke, blood oxygenation level dependent, functional magnetic resonance imaging, primary motor cortex, primary somatosensory cortex, cerebral palsy

## Abstract

Functional MRI is increasingly being used in the assessment of brain activation and connectivity following stroke. Many of these studies rely on the Blood Oxygenation Level Dependent (BOLD) contrast. However, the stability, as well as the accuracy of the BOLD response to motor task in the ipsilesional hemisphere, remains ambiguous. In this work, the BOLD signal acquired from both healthy and affected hemispheres was analyzed in 7-year-old children who sustained a Neonatal Arterial Ischemic Stroke (NAIS). Accordingly, a repetitive motor task of the contralesional and the ipsilesional hands was performed by 33 patients with unilateral lesions. These patients were divided into two groups: those without cerebral palsy (NAIS), and those with cerebral palsy (CP). The BOLD signal time course was obtained from distinctly defined regions of interest (ROIs) extracted from the functional activation maps of 30 healthy controls with similar age and demographic characteristics as the patients. An ROI covering both the primary motor cortex (M1) and the primary somatosensory cortex (S1) was also tested. Compared with controls, NAIS patients without CP had similar BOLD amplitude variation for both the contralesional and the ipsilesional hand movements. However, in the case of NAIS patients with CP, a significant difference in the averaged BOLD amplitude was found between the healthy and affected hemisphere. In both cases, no progressive attenuation of the BOLD signal amplitude was observed throughout the task epochs. Besides, results also showed a correlation between the BOLD signal percentage variation of the lesioned hemisphere and the dexterity level. These findings suggest that for patients who sustained a NAIS with no extensive permanent motor impairment, BOLD signal-based data analysis can be a valuable tool for the evaluation of functional brain networks.

## Introduction

With a birth-prevalence of approximately 1/5,000, neonatal Arterial Ischemic Stroke (NAIS) is identified as the most prevailing subcategory of the perinatal ischemic stroke (Dunbar and Kirton, 2018; Fluss et al., 2019). It is defined as a symptomatic cerebrovascular event between birth and 28 days of life with clinical or radiological evidence of focal arterial infarction (Fluss et al., 2019). For these patients, the middle cerebral artery (MCA) territory is the most commonly affected (Raju et al., 2007). The presence of clinical manifestation in the neonatal period is what differentiates NAIS from presumed perinatal stroke and venous infarction (Dunbar and Kirton, 2018).

Around 20–30% of full-term infants, with symptomatic NAIS, will develop cerebral palsy (CP; almost always unilateral spastic CP; Chabrier et al., 2016; Fluss et al., 2019). Motor impairment emerges typically in the first year, with a normal neurological examination until 4–6 months of age (Golomb et al., 2001). The resulting impairment is mostly unilateral due to the infarct site which is frequently located in the left hemisphere (Golomb et al., 2001). In cases of clinical impairment (developmental, motor and/or language) following NAIS, and more widely after pediatric stroke, functional brain analysis can be of great interest in the assessment of brain activation (Dinomais et al., 2015) and the understanding of the brain functionality, plasticity and (re)organization (Chen and Li, 2012). Accordingly, further knowledge about brain plasticity can help in planning precocious rehabilitation, re-education strategies and in targeting new approaches in rehabilitation fields.

In the last two decades, functional Magnetic Resonance Imaging (fMRI) has emerged as a useful neuroimaging technique in general (Chen and Li, 2012) and for children in particular (Herholz and Heiss, 2000; Fiori et al., 2018; Saunders et al., 2019) due to its non-invasiveness, absence of radiation exposure and good spatiotemporal resolution. Hence, it has become widely used in the brain mapping field as a tool to monitor and investigate brain neurophysiology and neuropathology (Chen and Li, 2012; Geranmayeh et al., 2015; Fiori et al., 2018). Notably, fMRI is one of the main imaging techniques used in the study of brain function and recovery after stroke (Krainik et al., 2005; Mazzetto-Betti et al., 2010; Bonakdarpour et al., 2015; van Oers et al., 2018). FMRI is based on the analysis of the Blood Oxygenation Level Dependent (BOLD) signal that indirectly reflects the variation in the neural activity through changes in the hemoglobin oxidative condition in the vascular bed encircling the activated neural tissue (Krainik et al., 2005; Bonakdarpour et al., 2015; van Oers et al., 2018). However, many studies have questioned the temporal stability (i.e.: variation of mean intensity through the different task epochs, e.g.: attenuation) and variability (i.e.: variation of the overall response shape, e.g.: time to peak) of the BOLD signal in the presence of lesions resulting from stroke (Mazzetto-Betti et al., 2010; Chen and Li, 2012; Bonakdarpour et al., 2015). Mazzetto-Betti et al. (2010) suggested a short-term attenuation of the BOLD signal in the lesioned hemisphere during fMRI motor tasks in patients with chronic ischemic stroke. Based on their findings, they advised researchers to use caution when performing BOLD analysis-based brain studies in stroke (Mazzetto-Betti et al., 2010). This might be relevant also for studying brain plasticity following strokes in a pediatric population. The temporal stability of the BOLD signal during fMRI motor activation, of the affected hand compared to the healthy one, likely depends on the associated residual motor function of the paretic hand. Accordingly, investigating the influence of the dexterity level on the variation of the BOLD signal amplitude during motor tasks might improve our understanding of the limitations and potential of BOLD fMRI after brain lesions.

In this work, we analyzed the BOLD signal stability during a motor task in a group of 7-year-old children that sustained a NAIS. We hypothesized that the stability of the BOLD-fMRI response would be preserved if the signal reflected the predicted fMRI response to the block design motor task performed by the subjects with no temporal or dynamic alterations. This was investigated by comparing ipsilesional with contralesional BOLD responses to see if there is a variation due to the lesion. Furthermore, differences between the intensities of the BOLD amplitude through the five motor task epochs were inspected for temporal stability (attenuation of intensities over time).

The precise timing, specific mechanisms, and locations of the unilateral lesion in NAIS provide an ideal model to study neuroplasticity after early brain lesions (Kirton, 2013). The aim of this study was first to test the stability of the BOLD signal amplitude in neonatal stroke, second to compare the BOLD response of both hemispheres for patients with and without CP (no CP = NCP), and lastly to explore the relationship between the BOLD signal intensity and the level of hand motor function.

## Materials and Methods

### Subjects

Patients belonged to the previously described cohort (Accident Vasculaire Cérébral du nouveau-ne, that is, neonatal stroke; PHRC régional n°80308052 and PHRC interrégional n°81008026; Eudract number 2010-A00329-30; Dinomais et al., 2015). One hundred term newborns with an arterial infarct, confirmed by early brain imaging (CT and/or MRI before 28 days of life), who were symptomatic during the neonatal period (Raju et al., 2007) were consecutively enrolled between November 2003 and October 2006 from 39 French centers. At the age of 7 years, a clinical follow-up visit was organized, and an MRI investigation was proposed to the 72 children who took part in this evaluation. Of the 52 children who participated in the MRI study, 38 had unilateral lesions in the MCA territory and constituted the patient population of this study (Eudract number 2010-A00976-33). Five participants were excluded from fMRI analysis due to head displacement during any movement greater than 3 mm or greater than 3° during each of the scan session. The final cohort was comprised of 33 patients with unilateral lesions in the MCA territory. Along with the NAIS patients we recruited 30 healthy controls with similar age and level of education. The general profile and characteristics of the participants are presented in Table 1 and a detailed description of the patients is presented in Table 2.

**Table 1:** General profile of the participants.

	Controls Mean (±SD) or *n* (%) *n* = 30	NCP Mean (±SD) or *n* (%) *n* = 23	CP Mean (±SD) or *n* (%) *n* = 10	*p*-values*
**Age (years)**	7.71 (±0.54)	7.40 (±0.34)	7.63 (±0.21)	0.461
**Gender**	Males: 14 (46.67%) Females: 16 (53.33%)	Males: 15 (65.22%) Females: 8 (34.78%)	Males: 7 (70.0%) Females: 3 (30.0%)	-
**Right-handed**	27 (90%)	14 (60.86%)	5 (50.0%)	-
**Lesion size (ml)**	–	18.9 (±23.03)	72.19 (±46.60)	0.0001
**TIV**	1395.4 (±110.01)	1443.7 (±150.68)	1313.5 (±74.25)	0.144
**BBT score**	–	31.8 (±6.55)	20.5 (±10.05)	0.0012
		33.4 (±7.75)	28.7 (±8.04)	0.0761

TIV, Total Intracranial Volume; CP, Cerebral Palsy; NCP, patients without Cerebral Palsy. *p-values are obtained by one-way Kruskal–Wallis nonparametric ANOVA.

**Table 2:** Demographic and clinical details of the Neonatal Arterial Ischemic Stroke (NAIS) patients evaluated in this study.

Patient No.	Handedness	Lesion side	BBT score		Lesion size (ml)	Cerebral Palsy (CP)
			Contralesional hand	Ipsilesional hand		
1	R	Left	N/A	N/A	30.65	No
2	L	Left	22	28	21.82	No
3	L	Left	44	42	35.48	No
4	R	Left	30	30	3.37	No
5	R	Left	28	25	38.39	No
6	L	Left	4	28	8.90	Yes
7	R	Left	39	29	0.29	No
8	L	Left	26	25	1.92	No
9	R	Left	28	31	48.26	No
10	L	Left	43	41	0.68	No
11	L	Left	30	40	84.98	No
12	R	Left	38	34	2.98	No
13	L	Left	29	34	23.92	No
14	L	Left	13	19	62.98	Yes
15	L	Left	37	42	31.11	No
16	L	Left	31	34	123.84	No
17	L	Left	40	42	69,07	Yes
18	L	Left	14	17	8.59	Yes
19	R	Left	31	35	19.28	No
20	R	Right	25	36	140.64	Yes
21	R	Right	25	31	34.34	No
22	R	Right	23	23	10.95	No
23	R	Right	19	24	42.55	Yes
24	R	Right	31	31	1.69	No
25	R	Right	26	34	83.81	Yes
26	R	Right	22	28	76.78	Yes
27	R	Right	22	31	127.43	Yes
28	R	Right	28	31	10.12	No
29	R	Right	33	33	2.93	No
30	R	Right	39	33	14.32	No
31	R	Right	28	34	15.07	No
32	R	Right	31	39	1.02	No
33	L	Right	27	44	0.65	No

*BBT, Box and Block test; M, Male; F, Female; L, Left-handed; R, Right-handed*.

As previously reported, the definition provided by the Surveillance of CP in Europe (SCPE) network was used (Cans et al., 2007). For the remainder of this text, we will refer to the two NAIS groups as CP (all unilateral) and NCP that denote patients with and without CP respectively.

Informed written consent respecting the declaration of Helsinki was obtained from all participants/parents as well as approval from the ethical committee of the university hospital of Angers, France. Handedness was determined according to the Edinburgh inventory (Oldfield, 1971).

### Manual Dexterity of Contra- and Ipsilesional Hands

In all children after NAIS, the motor performance of the ipsi- and contralesional hands was assessed with the Box and Block Tests (BBT), a reliable and validated tool for measuring gross manual dexterity in children (Mathiowetz et al., 1985). The individual subject’s score was defined as the maximum number of cubes transferred by the ipsi- and contralesional hand from one compartment to the other in 1 min.

### Functional MRI Acquisition and Processing

#### Acquisition

Functional MR images were acquired on a 3.0 Tesla scanner (MAGNETOM TrioTim system, Siemens, Erlangen, Germany, 12 channel head coil). An EPI sequence was used to acquire functional series (TR = 2,500 ms, TE = 30 ms, 40 axial sequential slices of 3.0 mm slice thickness, in-plane matrix = 64 × 64, yielding a voxel size of 3 × 3 × 3 mm^3^), covering the whole brain including the cerebellum. Sixty functional volumes were acquired per session.

#### Task Design

For functional MRI acquisition, the paradigm was implemented in block designs, with two alternate conditions: (1) the child was asked to repetitively open and close the hand at a frequency of 1 Hz (motor task condition). During this task, the child heard a 1 Hz metronome through standard MRI compatible headphone; and (2) the child was asked not to make any voluntary movement at rest (rest condition). The effective execution of the motor paradigm and the absence of actual hand movement during the rest period was controlled by the last author (MD) in a video recorded into the MRI channel during the fMRI sessions. The motor task was performed for 20 s followed by 10 s of rest. Each condition was repeated through five epochs. One session comprised 60 scans. Two identical and independent functional sessions were alternatively performed: one for right-hand movements and the second for left-hand movements. The experimental set-up was identical for the two motor sessions.

Before scanning, all the children underwent an fMRI training session using the hand movement paradigm inside a mock scanner that looks identical to the real scanner. The mock scanner was used to familiarize participants with the fMRI paradigm and the scanning environment. During this training session, the same investigator (MD) checked that subjects were able to follow the motor task design.

#### Functional Data Analysis

The functional data were processed using the Statistical Parametric Mapping software SPM12 (Department of Cognitive Neurology, London, UK^1^) running in Matlab 2017a (The MathWorks, Natick, MA, USA) on Ubuntu 18.04.2 LTS machine. Preprocessing of fMRI images included slice time correction, motion correction, and co-registration. All fMRI images were then smoothed with a Gaussian kernel of 8 mm FWHM and normalized to the Montreal Neurological Institute (MNI) template. These preprocessing steps were done in SPM12 using standard settings for fMRI.

A generalized linear model (GLM) was used for intra-subject analysis wherein a hemodynamic response function convolved with a boxcar function was used. The first-level analysis was applied for all subjects (controls and patients) to obtain the fMRI statistical maps relative to both right and left-hand activation (Motor task). Next, second-level statistical analysis with a GLM was performed to obtain a common activation region for both hands. Furthermore, a parametric GLM based on the one proposed by Mazzetto-Betti et al. (2009, 2010) was tested. It consisted, in addition to the classical hemodynamic response function regressor, of a second regressor that includes a time modulated hemodynamic function that describes a progressive attenuation. All statistical maps were corrected for multiple comparisons using Family Wise Error (FWE) with a threshold set to *P* < 0.05. In the case of parametric GLM, no cluster survived the FWE correction for nearly any of the subjects.

### Definition of Regions of Interest (ROIs)

Region of Interest (ROI) analysis was performed to investigate the BOLD response to the motor task. This included the analysis of the BOLD response for both ipsi- and contralesional hands. In this study, two different approaches were used for the definition of ROIs for the two sessions (left and right-hand movement). The first was based on the identification of activated regions in the control group (van Oers et al., 2018) and the second consisted of using predefined ROI templates. The two types of ROIs were included in this study to test the accuracy of each method of definition in the extraction of the BOLD response during the motor task and thus find the most efficient ROI that correctly describes the activated area.

**Figure 1:**
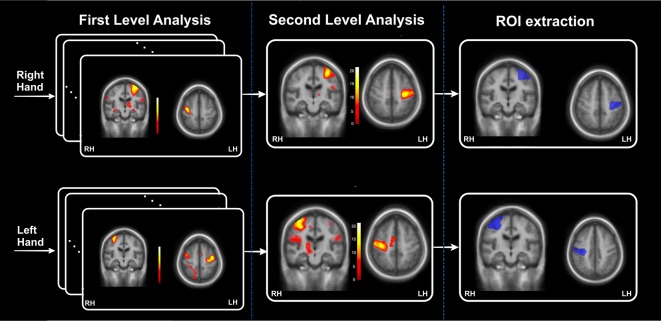
The block diagram relative to the definition of activation regions of interest (ROIs) extracted from the control’ statistical maps. This block diagram is divided into three parts: a first-level statistical analysis applied for each of the individual functional magnetic resonance imaging (fMRIs) of the 30 controls, second-level analysis to obtain the activation statistical maps and finally extraction of the activation ROIs as the cluster with the maximum *t*-values.

For the definition of activation ROIs, we used the statistical maps obtained from the second-level analysis (one-sample *t*-test) conducted on the control group. This analysis was performed on the signal contrast corresponding to the motor task vs. the rest condition for the two sessions. These, activation maps (FWE corrected, *P* < 0.05) reflected areas activated during left and right-hand movement in the control group. Afterward, we used the MarsBAR (MARSeille Boîte À Région d’Intérêt) toolbox of Brett et al. (2002) to extract and define one ROI for each task. The block diagram of the activation ROIs definition is presented in Figure 1. The ROI relative to each motor task was chosen from the cluster with the maximum *t*-values as indicated in Figure 1. These ROIs are referred to in this study as M ROIs. In the NAIS patients, the first-level analysis revealed clusters around the same location as the ROIs obtained from controls but much smaller in size. However, these clusters did not survive FWE correction. Consequently, the activation ROIs obtained from the control group were used for all the subjects.

**Figure 2:**
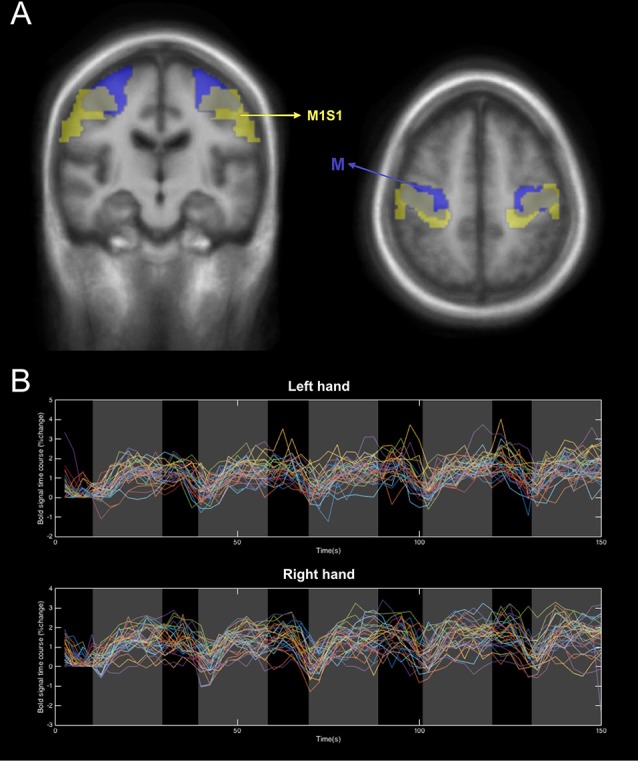
An illustration of the two types of ROIs used in this study. **(A)** The Atlas-based ROIs, M (yellow) and the activation ROIs, M1S1 (Blue). **(B)** An example of the BOLD signal extracted from the M ROI of the control group during activation of left and right hands.

For the Atlas-based ROIs, we used the WFU_PickAtlas toolbox (Maldjian et al., 2016) to extract the freely accessible ROI templates of the Primary Motor Cortex (M1) and the primary somatosensory cortex (S1). These regions were chosen as representative of the activated areas during the predefined motor tasks. Thus, the M1 ROIs were extracted from the Broadmann Area (BA) 4 for the LH and RH. As for S1, we included the three areas BA 1, 2 and 3 in the ROIs masks. Subsequently, we chose to combine the two regions M1 and S1 for each hemisphere in one global ROI that we called M1S1. These ROIs are presented in Figure 2A along with the activation ROIs.

The lesion masks for the patients were obtained from a recent study using the same database (Dinomais et al., 2015). In this study, the authors manually delineated the lesion slice by slice using 3D T1 MRI images to create binary individual lesion masks (Dinomais et al., 2015). The sum of these normalized binary lesion masks is shown in Supplementary Figure SA. By inspecting both Figure 2A, we can detect a possible overlap between the lesion and the ROIs (M and M1S1) depending on the lesion location. This is the case for some of the patients, especially those with bigger lesion volumes. Based upon this, the lesion mask for each patient was removed from the ROI cluster to obtain an accurate BOLD signal that reflects activation from the brain motor system and not from cerebrospinal fluid inside the infarct (Schreiber et al., 2000). This step was important in the analysis because, in the contralesional hand movement of patients, some lesions overlap with the cluster used to compute the BOLD response. This can lead to an inaccurate representation of the %BOLD response to the task by adding voxels of cerebrospinal fluid and then averaging the corresponding signal with the rest. As is known, the response extracted from voxels in the cerebrospinal fluid does not portray any variation concerning the task and thus it acts as a constant noise (Schreiber et al., 2000). Accordingly, it was important to remove this noise considering it will only cause the overall amplitude of the % BOLD response to drop. Nevertheless, we have to point out that the voxels removed from the ROI of each patient were less than 10% of the total ROI volume. Consequently, removing them did not impact the signal to noise ratio of the extracted BOLD signals.

### BOLD Signal Extraction and Processing

The BOLD signal percentage change for each subject was obtained as follows: for each ROI (M and M1S1) and each session (left and right-hand activation), the time series were computed as the mean signal of all the voxels inside the ROI cluster. Afterward, these signals were converted into the %BOLD signal time series by scaling each time point by a baseline. We have to point out here that the chosen rest period was very short (10 s). This does not allow the signal sufficient time to attain rest before the next stimulation. For this reason, the resting baseline was determined as the average of the bold signal throughout the first resting period before the beginning of the activation. For a detailed description of the computing algorithm please refer to the Supplementary Document (Section A). Then, the BOLD signals corresponding to the movement of the ipsilesional (unaffected hand) and the contralesional (affected) hand were grouped (i.e.: for the patients with a lesion in the RH, the affected hand was the left hand and vice versa). An example of the resulting % BOLD responses for the left and right-hand movements averaged across control subjects are presented in Figure 2B.

To compute the average %BOLD response per task epoch, we averaged the %BOLD response values in a 10 s window after 10 s of hand movement to avoid the lag time to the peak period (for further details please refer to the Supplementary Document Section SB and Supplementary Figure SB).

### Statistical Analysis

Statistical tests across groups and tasks were conducted using Matlab 2017a. For the comparison between healthy and patient groups and between ipsilesional and contralesional hand movement responses, a two-sample *t*-test was used. A Kruskal–Wallis test was performed for the comparisons between CP and non-CP (NCP) groups since Kolmogorov-Smirnov tests revealed that the data was not normally distributed. Finally, for the correlation analysis, Spearman’s correlation coefficient was used. All results with *p* < 0.05 were considered significant.

## Results

**Figure 3:**
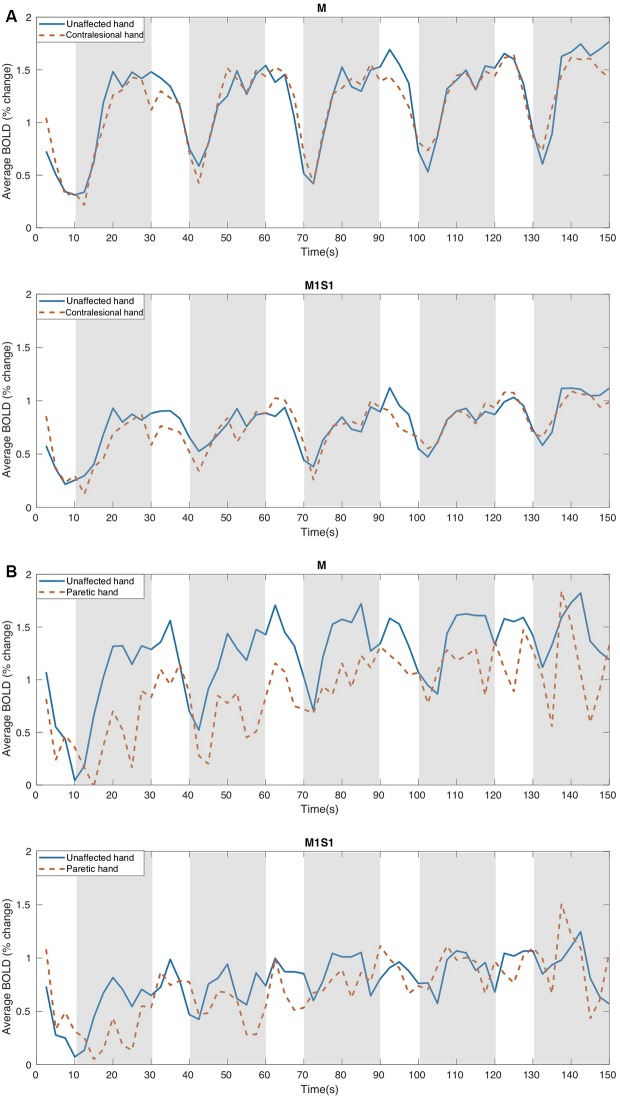
The Mean % Bold variation course for: **(A)** NCP (no cerebral palsy) and **(B)** CP patients using the two types of ROIs, M, and M1S1, in response to the motor task performed with the unaffected and the contralesional (paretic in the case of CP) hand. M indicates the ROIs obtained from the controls activation areas and M1S1 represent the atlas-based ROIs. The motor task intervals are illustrated in gray.

**Figure 4:**
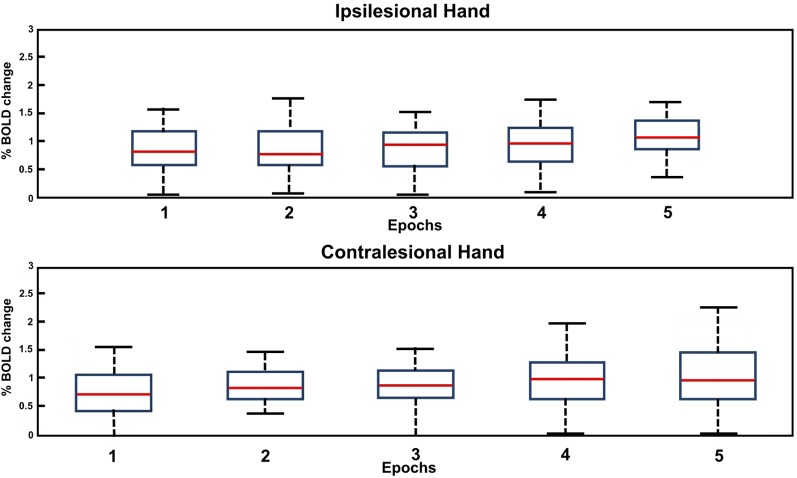
Box plot of the BOLD amplitude variation through the five epochs of the motor task for the affected (contralesional) and unaffected (ipsilesional) hand in the case of NCP for the M ROI.

Figure 3 presents the % BOLD response to the motor task executed with each hand for M and M1S1 averaged for NCP (Figure 3A) and CP (Figure 3B) groups. The corresponding mean and SD values are displayed in Table 3. For the NCP patients, no significant difference was observed between the % BOLD change for the ipsilesional and the contralesional hand for both M and M1S1. The mean %BOLD response was higher in M (Figure 3) compared to M1S1 for both the NCP and CP groups. This suggests that M depicts a more accurate representation of the activated region during the movement of each hand than the atlas-based ROI M1S1. Furthermore, the BOLD signal response for both the ipsilesional and the contralesional motor task was found to be stable throughout the five epochs as depicted in Figure 4 for the M ROI.

**Table 3:** The mean BOLD Signal (percent change) in response to the motor task performed with the contralesional and ipsilesional hand averaged across the five epochs.

		Ipsilesional hand Mean ± SD	Contralesional hand Mean ± SD	*p*-value
NCP	M	1.46 ± 0.53	1.37 ± 0.55	0.492
	M1S1	0.90 ± 0.40	0.84 ± 0.48	0.625
CP	M	1.41 ± 0.57	0.96 ± 0.53	0.048
	M1S1	0.83 ± 0.56	0.72 ± 0.42	0.508

For the CP group, when comparing the BOLD amplitude variation in M, a clear dissimilarity can be observed between the unaffected and the paretic hand. In the case of the unaffected (ipsilesional) hand, a stable signal course was depicted through all the epochs, whereas for the paretic (contralesional) hand the mean BOLD profile seems to be less steady with lower values throughout the five epochs (see Figure 3B). In this case, we can distinguish the block design pattern of the command but with decreased % BOLD variation for the paretic hand compared to the unaffected one. The comparison of the % BOLD variation averaged across the epochs revealed a significant difference between the paretic and the unaffected hand in the M area (see Table 3). On the other hand, the average % BOLD response extracted from M1S1 did not depict a comparable trend. Both BOLD responses were not stable enough to reflect the activation response to the motor task. Similarly, *t*-tests did not reveal a significant difference between the two signals (Table 3). Based on these results, we can assume that the M1S1 ROI does not accurately reflect the activated voxels associated with the motor task performed by the subjects when using their paretic hand. Thus, for the remainder of this study, only the BOLD signal obtained from activation ROI (M) will be considered.

It should be noted that no progressive attenuation of the % BOLD response was detected for any of the patients (CP and NCP) during motor task completion with the contralesional hand. This was observed in Figure 3 for the mean BOLD amplitude of NCP and CP groups and was confirmed by inspection of the individual BOLD time course of each patient for the M and M1S1 ROIs. The boxplot of the bold variation through the five epochs for each hand is depicted in Figure 4 for the NCP group.

**Figure 5:**
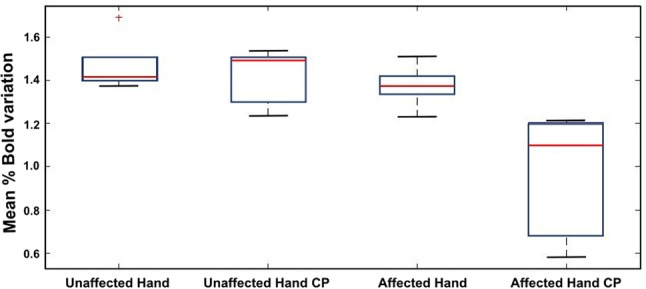
Comparative study of the mean amplitude of the BOLD response to the affected vs. unaffected hand motor task in the case of NCP and CP patients.

To further investigate the BOLD signal trend and to compare between the two groups (NCP and CP), we performed a Kruskal–Wallis test on the BOLD signal amplitude of the NCP and CP groups for both hand activation tasks averaged across the five epochs. The results are depicted in Figure 5. By analyzing the box plot in Figure 5, we can notice the low values obtained for the paretic hand of the CP group. *Post hoc* tests revealed a significant difference between the paretic (contralesional) and unaffected (ipsilesional) hand for the CP group (*p* = 0.0086, *X*^2^ = 11.67) and between the paretic hand of CP group and both ipsi- and contralesional hands of NCP group.

**Figure 6:**
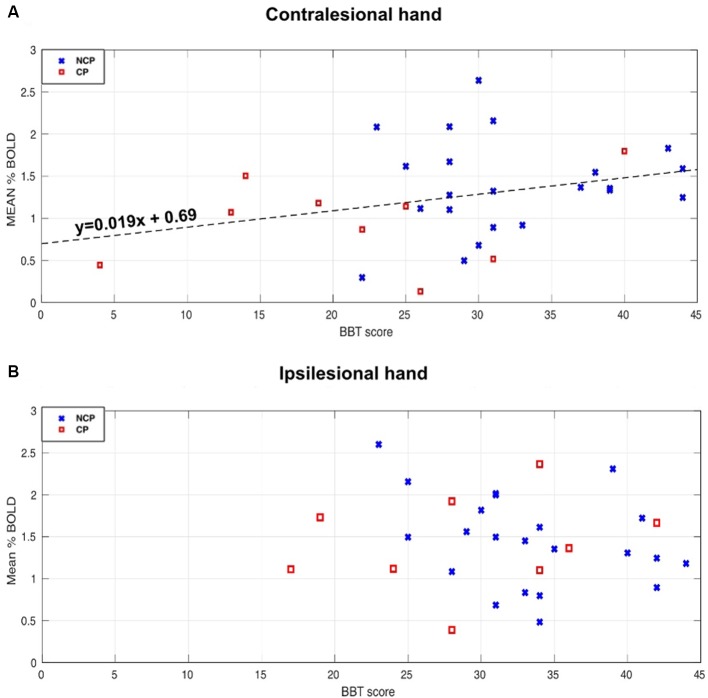
Relationships between Bold signal percentage change and Box and Block Tests (BBT) score for the NCP (blue X) and CP (red square) subjects. **(A)** Scatterplot of the contralesional BBT score and the mean BOLD amplitude of the lesioned hemisphere when activating the contralesional hand. A significant positive correlation (rho = 0.33, *p* < 0.05) was found for both groups (CP and NCP). The Dashed line represents the linear fitting for all the subjects. **(B)** The relationship between the ipsilesional BBT score and the mean BOLD amplitude of the unaffected hemisphere when activating the ipsilesional hand. No significant correlation was found (rho = −0.14, *p* = 0.45).

Figure 6 demonstrates the correlation between the BBT score of each hand and the corresponding contralateral % BOLD signal response for CP (red) and NCP (Blue) patients averaged across the five epochs. For the contralesional hand, a positive correlation between the BBT and the BOLD percentage change was revealed by statistical tests (Spearman’s rho = 0.33, *p* = 0.0026; Figure 6A). This indicates that the higher the BBT score obtained by the patient for the contralesional hand motion, the higher the BOLD response of the lesioned hemisphere, independently of the lesion size and the presence of CP. Figure 6B depicts the relationship between the BBT score of the ipsilesional hand and the mean BOLD amplitude relative to the healthy (unaffected) hemisphere. No significant correlation was found between the ipsilesional BBT score and the mean % BOLD signal (CP and NCP; rho = −0.14, *p* = 0.45).

## Discussion

Even though it has been suggested by several studies that the BOLD response can be unreliable in the case of stroke (Sakatani et al., 2007; Mazzetto-Betti et al., 2010), researchers continue to use it as a noninvasive tool to measure different outcomes and characteristics of the lesioned brain (Butler and Page, 2006; Chau et al., 2010; de Haan et al., 2013; Khalil et al., 2017). In this work, we have investigated the functional response of 7-year-old children, who have suffered a unilateral neonatal stroke, to a simple motor task performed by both contra- and ipsilesional hands. Our findings suggest that the BOLD signal can be a reliable tool for investigating different outcomes after NAIS. This can be of great interest in the evaluation of different interventions and rehabilitation techniques on particular brain functions following neonatal stroke (Rossini et al., 2003).

One parameter that should be chosen with great care is the ROI used for extracting the fMRI-BOLD signal (Logothetis, 2002). It has been demonstrated that the BOLD contrast variation depends on the brain’s structural, physical, topographical and ultrastructural properties (Schreiber et al., 2000). In the case of stroke, the lesion in the affected hemisphere occupies a certain space that contains glial cells that alter the BOLD contrast (Schreiber et al., 2000). Based on the above, the two different types of ROIs used in this study to extract the BOLD response revealed that activation-based ROIs (obtained from the control group) allows for a more accurate fMRI response compared to atlas-based motor region ROIs. This was demonstrated in Figure 3 where we obtained higher % variation as well as more stable profiles for the activation-based ROIs. The difference between the two obtained BOLD variations could be due to the location difference between M and M1S1 (Figure 2A), where M1S1 seems to encompass a more lateral area of the brain (including more areas of the motor homunculus) whereas the M ROI was more closely constrained to the hand knob area. We have to emphasize here, that the two types of ROIs that were used in this work did not contain any voxels belonging to the lesion site.

Our analysis of the % BOLD response stability through all five stimulation epochs revealed no progressive attenuation in either hand regardless of having CP or not (please refer to Figures 3, 4). In other studies with the block design, the BOLD signal following stroke exhibited progressive attenuation through task epochs (Mazzetto-Betti et al., 2010; Veldsman et al., 2015). This is attributed to the fact that stroke patients may undergo muscular fatigue or weakness when faced with multiple repetitive tasks (Mazzetto-Betti et al., 2010). However, the fatigue hypothesis was formulated in the case of adult stroke (Snaphaan et al., 2011; Storti et al., 2014) and may not necessarily apply in the case of neonatal stroke.

In our study, the parametric GLM used to test the attenuation phenomenon, failed to determine the activation areas relative to the motor task (no cluster survived the FWE correction). Therefore, it seems that the hypothesis of progressive attenuation of the BOLD signal response to contralesional hand movement does not apply in the case of NAIS survivors with and without CP.

We also compared the BOLD responses in the contralesional and ipsilesional hemispheres during hand movements for CP and NCP patients. For the NCP group, we obtained similar trends and amplitude variation of the BOLD signal for both ipsi- and contralesional hands (see Table 3). This can be explained by the normal motor performance exhibited by these patients as measured by the BBT score of the contralesional hand compared to average values (see Table 2). In contrast, for the CP group, lower BOLD variation values were observed for the paretic hand (see Table 3), i.e., in the lesioned hemisphere. Furthermore, a Kruskal–Wallis test comparing all four subgroups (Figure 5) revealed a significantly lower BOLD percentage change during paretic hand movement in the CP group compared to the unaffected (ipsilesional) hand of both CP and NCP groups and the contralesional hand of the NCP group. This lower BOLD response variation for the affected hand in the CP group can be explained by different hypotheses. The first one concerns the size and location of the lesion concerning the activated region as well as to the variability due to plastic changes. The lesion size is higher for CP patients (see Table 2). There is also more overlap with the activation ROI, which means that fewer voxels are retained for the BOLD signal extraction. Therefore, the remaining voxels may not represent the primary voxels that would be activated if the lesion did not exist. Another explanation can be associated with altered neurovascular coupling. Few studies have pointed out a decreased BOLD amplitude in activation regions when performing a motor task with the ipsilesional hemisphere (Pineiro et al., 2002; Krainik et al., 2005; Murata et al., 2006). This implies an impairment in the cerebrovascular reactivity of patients with CP in our case. Another hypothesis could be that the hand movement in the case of paretic hand for some patients is highly limited. Therefore, the signal extracted from the remaining voxels does not represent a normal response to the motor task command. We think that the latter hypothesis seems the most plausible since some of the CP patients suffered from considerable impairment (BBT < 10).

Our study examined the relationship between the BOLD signal and the motor performance of the patients. We found a positive correlation between the BBT score of the contralesional hand and the average amplitude of the lesioned hemisphere BOLD signal regardless of having CP or not (please refer to Figure 6A). This confirms that the BOLD response is associated with the motor performance level of the patients. However, no significant correlation was found between the BBT score of the ipsilesional hand and the corresponding mean % BOLD response. This can be explained by the small variance of the BBT score between patients for the unaffected hand. Another explanation is that the BOLD signal does not accurately reflect the functional activation in those with severe impairment (very low BBT score). Nevertheless, for CP patients with a high BBT score (>25), the BOLD signal corresponding to the paretic hand motor task was stable throughout the five epochs. Based on these observations, it seems that the BOLD signal intensity depends on the motor performance and dexterity of the patients more so than the fact that they sustained a CP. This is in line with previous findings by Thébault et al. (2018), where they demonstrated that manual dexterity was the primary criterion of cognitive functioning, regardless of the CP disorder that existed.

The results presented in this study point to the importance of the BOLD response as a tool for functional activation analysis in children after NAIS. It can be used for different application such as rehabilitation for evaluating the effectiveness of the different approaches for recovery (Rossini et al., 2003), neuroplasticity modeling for assessing reorganization patterns (Ward and Cohen, 2004) and predicting different outcomes through noninvasive measures (Lake et al., 2016).

We need to point out some limitations of this work. A particular disadvantage was concerning the block design paradigm used for the fMRI acquisition, where the sequence was short, but the rest period was brief (10 s) following the stimulation stage (20 s). This rest period might be insufficient for the signal to return to baseline which can contribute to a BOLD drift. In our case, however, the BOLD drift was remedied by scaling the extracted signals for the first rest epoch and by computing the average % signal change of the BOLD in the second half of the stimulation period (last 10 s of hand activation task) where the signal was stable. There was also inhomogeneity in the number of patients in the CP (*N* = 10) and NCP (*N* = 23) groups which leads to different statistical powers.

Another limitation is the use of activation ROIs extracted from healthy controls that do not accurately represent the motor area in the case of very impaired children that may have gone through functional reorganization following the stroke. Lastly, although the results might apply to other types of lesions, especially cortical lesions in children, the findings presented here are specific to children after neonatal stroke. Further investigations will be needed to extend the results to other lesions at different chronological ages.

## Conclusions

Although the BOLD signal stability has been questioned in the literature for patients after stroke (Pineiro et al., 2002; Rossini et al., 2004; Mazzetto-Betti et al., 2010; Chen and Li, 2012), the findings presented in this study suggest otherwise in the case of NAIS survivors. Based on our results, the BOLD signal response (in the lesioned hemisphere) during the contralesional hand movement seemed to be stable in patients regardless of the presence of CP. Furthermore, the % BOLD response to contralesional hand movement appears to be correlated to the motor performance (BBT score) for both CP and NCP groups. Another important element is that no progressive attenuation of the BOLD signal was observed for either case. This implies that the conventional GLM model analysis can be used without extensive caution. Finally, we conclude that the BOLD signal can be a valuable tool in brain functionality investigation after neonatal stroke.

## Data Availability Statement

The raw data supporting the conclusions of this article will be made available by the authors, without undue reservation, to any qualified researcher.

## Ethics Statement

The studies involving human participants were reviewed and approved by University Hospital of Angers. Written informed consent to participate in this study was provided by the participants’ legal guardian/next of kin.

## The AVCnn Study Team

Stéphane Darteyre, MD, PhD (Univ Saint-Étienne, data collection and analysis); Céline Dégano, MSc (Univ Saint-Étienne, data collection and analysis); Matthieu Delion, MD, PhD (Univ Angers, data collection and analysis); Johanna Deron, MSc (CHU Saint-Étienne, language assessment); Gérard Dray, PhD (Mines Alès, statistical analysis supervision); Laure Drutel, MSc (CHU Saint-Étienne, language assessment); Samuel Groeschel, MD (University Children’s Hospital Tübingen, data collection and analysis); Lucie Hertz-Pannier, MD, PhD (CEA, imaging study); Béatrice Husson, MD (CHU Bicêtre, imaging study); Manoëlle Kossorotoff, MD, PhD (Hôpital Universitaire Necker-Enfants Malades, data collection and analysis); Leila Lazaro, MD (CH Côte Basque, data collection); Jérémie Lefranc, MD (CHU Brest, data collection); Sylvie Nguyen The Tich, MD, PhD (CHU Lille, study design and data collection and analysis); Emeline Peyric, MSc (CHU Saint-Etienne, cognitive assessment); Emilie Presles, MSc (CHU Saint-Etienne, statistical plan); Magaly Ravel, MD (Univ Saint-Étienne, data collection and analysis); Cyrille Renaud, PhD (CHU Saint-Étienne, study coordinator and data collection and analysis); Guillaume Thébault (MsC, University of saint étienne); and Carole Vuillerot, MD, PhD (CHU Lyon, data collection and analysis).

## Author Contributions

All authors contributed to the conception and design of the study as well as the writing of the manuscript. All authors contributed to manuscript revision, read and approved the submitted version.

## Conflict of Interest

The authors declare that the research was conducted in the absence of any commercial or financial relationships that could be construed as a potential conflict of interest.

## Footnotes

^1^https://www.fil.ion.ucl.ac.uk/spm/software/spm12/
