# Supplementary material for: Is the Blood Oxygenation Level-Dependent fMRI Response to Motor Tasks Altered in Children After Neonatal Stroke?
Source: Front Hum Neurosci. 2020 Apr 29;14:154. doi: 10.3389/fnhum.2020.00154 (PMC7202247; doi:10.3389/fnhum.2020.00154)
Supplement: Supplementary file 1 [file Table_1.DOCX]

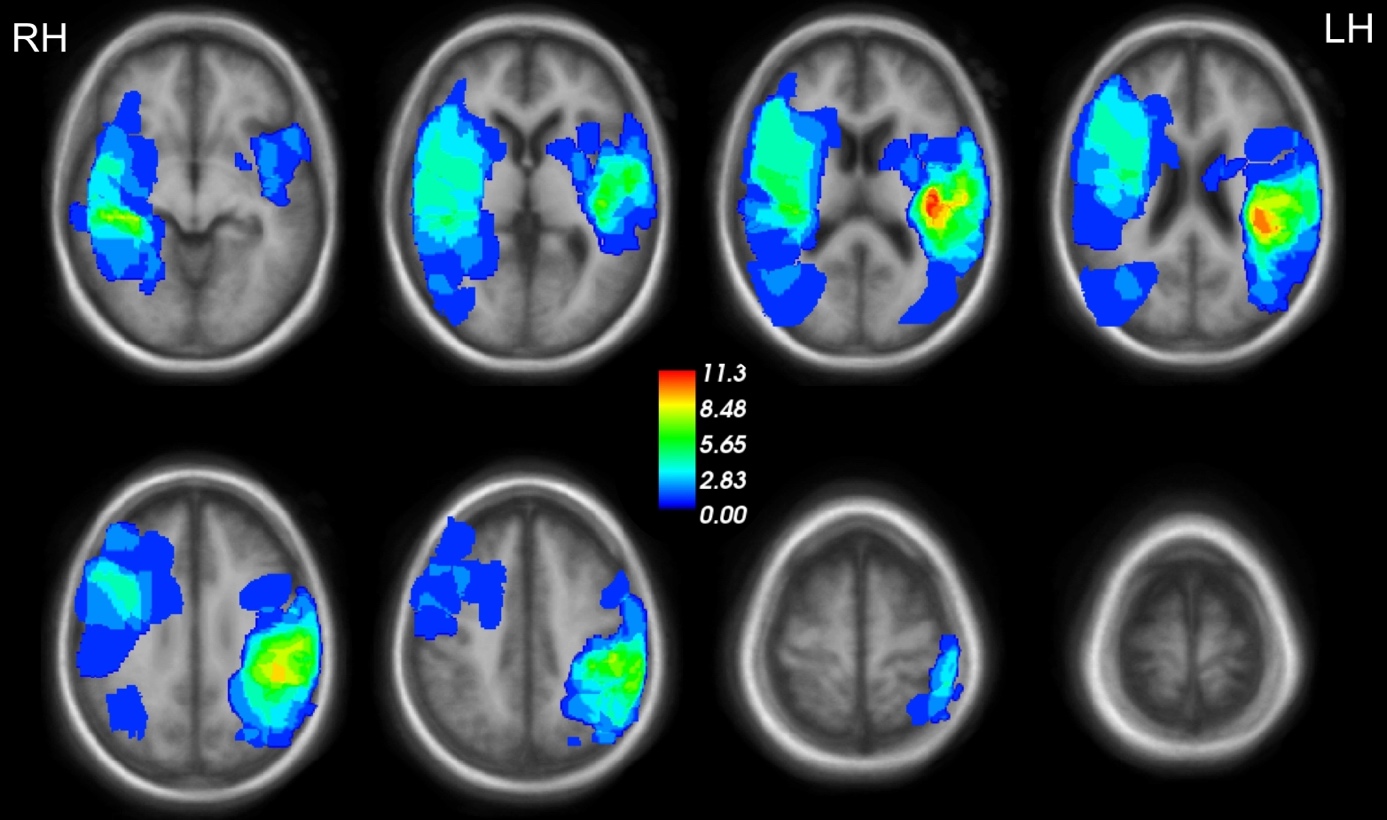


**Supplementary Figure A:** The group lesion masks for the NAIS patients.

1. **Computing the percent signal change (% BOLD response)**

In order to compute the signal percent change for each subject and for each hand activation task (left and right), first we computed the average signal of all the voxels in the ROI (obtained from activation ROIs or from atlas-based ROIs, please refer to section ***2.3. Definition of Regions of Interest (ROIs)***). We have to add here that in the case of NAIS patients the voxels that overlapped with the patient’s lesion map were excluded from the ROI and hence from the signal averaging calculation. If we consider the averaged signal X(t), the %BOLD Y(t) was computed as follow:

$\boldsymbol{Y}\left( \boldsymbol{t} \right)\boldsymbol{=}\frac{\boldsymbol{X}\left( \boldsymbol{t} \right)\boldsymbol{-B}}{\boldsymbol{B}}$ **(1)**

Where B is the baseline value specific to the subject, this baseline was obtained by:

$\boldsymbol{B=}\frac{\sum_{\boldsymbol{t=1}}^{\boldsymbol{10}} \boldsymbol{X(t)}}{\boldsymbol{10}}$ **(2)**

1. **Computing the mean percent signal change (average % BOLD response)**

In the group comparison analysis (between contra and ipsi-lesional hand movement, and CP/ NCP patients) as well as for the correlation study between the BBT and the % BOLD response we computed the average %BOLD response across the five epochs this was obtained by computing first the average of the signal Y(t) in a 10s window after 10s of activation to avoid the lag time to the peak as shown in (Figure B Bellow). Accordingly, the average values are obtained as follow:

$\boldsymbol{z}\left( \boldsymbol{k} \right)\boldsymbol{=}\frac{\sum_{\boldsymbol{t=10\times(3}\boldsymbol{k-1)}}^{\boldsymbol{10\times3}\boldsymbol{k}} \boldsymbol{y(t)}}{\boldsymbol{10}}$ **(3)**

Where k is the Epoch number (1 à 5)


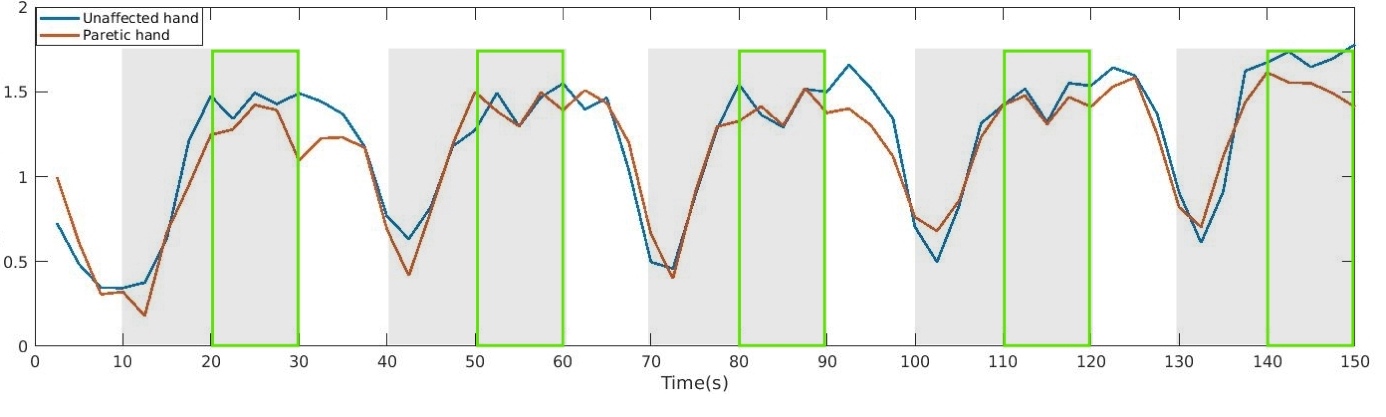


**Supplementary Figure B:** The windows used to compute the average %BOLD through the five activation epochs.
